# Integrated geochemical and magnetic potentially toxic elements assessment: a statistical solution discriminating anthropogenic and lithogenic magnetic signals in a complex area of the southeast Nile Delta

**DOI:** 10.1007/s10661-024-12408-5

**Published:** 2024-02-16

**Authors:** Alshymaa Mohammad Guda, Ahmed Mohamed El Kammar, Hend Saeed Abu Salem, Atef Mohammady Abu Khatita, Mohamed Abdelwahed Mohamed, Ibrahim Aly El-Hemaly, Esmat Mohamed Abd Elaal, Hatem Hamdy Odah, Erwin Appel

**Affiliations:** 1 Earth’s Geomagnetism Lab, National Research Institute of Astronomy and Geophysics (NRIAG), P.Box:11421, Helwan, Egypt; 2 Geology Department, Faculty of Sciences, Cairo University, Giza, Egypt; 3 Geology Department, Faculty of Sciences, Al-Azhar University, Cairo, Egypt; 4 Geology Department, College of Science, Taibah University, Taibah, Saudi Arabia; 5 Department of Geosciences, Tübingen University, Tübingen, Germany

**Keywords:** Toxic elements, Magnetic proxy, Complex setting, PCA, Cluster analysis, Nile Delta

## Abstract

Magnetic proxy approaches proved to be efficient for potentially toxic elements (PTEs) pollution assessment when targeting forests or areas with a homogenous background where anthropogenic magnetic signals could be easily distinguished. Here, we present a multidisciplinary approach for magnetic susceptibility ($x$) and HM assessment in a complex area in the Nile Delta, where geogenic input, land use, and various industries with different fly ash and surface water emissions interfere. Statistical analysis discriminates between the effects of lithologic elements and the concentrations of toxic anthropogenic elements. The studied elements are classified into lithogenic and anthropogenic-related (HMs, Au industry, and fertilizers industry) groups with maximum contamination levels of eight anthropogenic-related and highly toxic PTEs (Cu, Zn, Mo, Cd, Sb, Pb, Hg, and As) in the Akrasha industrial area (pollution load index = 15.84). Considering the whole data set, the numerical correlation of $x$ with most PTE concentrations and the pollution load index (PLI) is weak, while it is moderate to strong with lithogenic elements. However, a comparison of lithogenic elements and PTE concentrations along with *x-*values in two separate clusters supports the correspondence of lithology with elevated *x*-values in silt and clay-rich soil samples as well as HM concentration in industrial sandy soils. Correspondence between magnetic maps and chemistry data with land use reflects the potential of magnetic proxy methods for qualitative PTE pollution pre-delineation of the polluted spots, provided that lithological conditions are carefully considered.

**Supplementary Information:**

The online version contains supplementary material available at 10.1007/s10661-024-12408-5.

## Introduction

Soil is a non-renewable vital resource for life that requires to be sustained through periodic assessment for pollution risk detection. Heavy metals are common non-degradable potentially toxic elements (PTEs) (Pourang et al., 2005) that can harm vital organs and cause carcinogenicity (Sobhanardakani, 2017). Monitoring pollution levels is crucial for controlling toxicity, protecting human health, and ensuring Earth’s system sustainability. Magnetic susceptibility measurements are potentially effective tools for PTE pollution detection. The high sensitivity of the magnetic signal toward minute amounts of ferrimagnetic Fe-oxides (~ μg/kg in equivalent chemical analyses) in soil encouraged researchers to use magnetic measurements to get information about the soil mineralogy and composition (Blundell et al., 2009; Petrovský et al., 2000; Spiteri et al., 2005). Fe oxides are formed naturally in the soil through weathering and pedogenic processes, while those produced by different industries, through fly ash emission, transportation, and deposition on soils, are genetically related to HMs (Wang et al., 2021). Heavy metals tend to be absorbed, adsorbed, co-precipitated, or form complexes with Fe-oxides and hydroxides (Awofolu et al., 2005; Mohiuddin et al., 2010; Okafor & Opuene, 2007). Magnetic proxy tools are rapid and more economical compared to chemical analysis. The sensitivity of magnetic parameters to reflect heavy metal pollution levels makes them commonly used in pollution assessment research worldwide. However, they are rarely used in the Middle East, especially in Egypt, where very few studies were published (e.g., Saleh et al., 2022; Shetaia et al., 2022; and Guda et al., 2020).

Egypt’s leading agricultural, domestic, and industrial province is the Nile Delta. As a result, it is the most polluted sector with high toxicity levels of PTEs. Magnetic-based pollution studies often avoid the effect of land use on the soil magnetic signal by targeting forest soils, where deposited anthropogenic magnetic particles are well preserved in the relatively undisturbed topsoil (Anis et al., 2023; Magiera et al., 2007). Even more, studies in such areas were able to characterize magnetic particles from different industries. They were found to be significant for airborne particles from organic fuel combustion, mining, metallurgical, and human-related activities (Blaha et al., 2008; Magiera et al., 2023). Unfortunately, the Nile Delta heterogeneous land use impedes such types of studies and limits their significance. In addition to agriculture, various urban and industrial activities were introduced after the High Dam construction in 1964 which stopped the annual soil renewal in the Nile Delta causing a continuous accumulation of industrial pollutants. The subsequent lack of soil fertility led to the excessive use of fertilizers and pesticides. Industrial, agricultural, and residential activities are greatly interfering and produce enormous amounts of untreated waste that go directly into the Nile River (Shakweer & Yousef, 2011). The Nile Delta comprises 35 industrial zones with different industries (e.g., chemical, metallurgical, cosmetics, textiles, food, beverage). The emitted PTEs are transported downwind as airborne particles and deposited on the topsoil. Irrigation and other human activities transport these pollutants through water bodies to the topsoil, with detrimental impacts on humans and the ecosystem. HM data of the industrial effluents in the Delta region indicated average concentrations of 0.013–0.025, 0.03, 0.05, and 0.01 mg/L for Cd, Cu, Zn, and Pb, respectively (Fleifle & Allam, 2016). The published studies considered the Nile Delta, mostly targeting groundwater and/or its quality (e.g., Abdo, 2006; Abo El-Magd et al., 1999; Abu Salem et al., 2021), while those addressing the area’s soil focused on a certain pollution spot or a factory (e.g., Awadallah lead smelter and Abu Zaabal fertilzer factory; Ali et al., 2011). Few publications, preceding this study, documented HM concentrations in the Delta’s soil (e.g., Abu Khatita, 2011; Khalifa & Gad, 2018), and little is known about the significance of magnetic susceptibility to reflect HM pollution within such interfering complex situation (Delbecque et al., 2022).

Guda et al. (2020) studied the spatial and historical distribution of magnetic signals in the Nile Delta. They concluded that magnetic proxy data could identify the spatial distribution of the main pollutive industrial spots where magnetic parameters agreed, to a great extent, with HM downcore distribution. Moreover, Guda et al. (2020) raised the problem that weak to moderate correlations detected for magnetic properties with HM concentrations within cores may be attributed to different lithologic compositions.

In this study, we aim to provide a comprehensive geochemical analysis to delineate pollution sources, PTE distribution, and their possible risks. We also aim for clarification of the complex magnetic signal from anthropogenic and pedogenic sources in the light of geochemistry data. This study complements the one by Guda et al. (2020), which proved the constrained viability of magnetic proxies to reflect HM pollution in such a complex setting, mainly due to lithologic effects. We made use of magnetic susceptibility (*x*) data and HM downcore distributions for more reliable geochemical analysis and better translation of the magnetic signal complexity. For this, we applied advanced statistical analyses to distinguish the effects of both lithology and HM concentration on *x*, which should be considered when magnetometry is used as a preliminary assessment tool in such areas.

## Materials and methods

### The study area

Abu Zaabal, and its vicinity, is known as one of the most polluted areas in the Nile Delta. The area is located southeast of the Nile Delta between latitudes 30° 13′ 57.37″ and 30° 19′ 51.17″ N and longitudes 31° 19′ 15.16″ and 31° 24′ 10.84″ E (⁓80 km^2^). El-Shazly et al. (1975, 1978) and RIGW (1988) discussed the geology of the eastern part of the Nile Delta. The area is mainly covered by sediments from Pleistocene to Holocene ages. These sediments are represented by sand, silt, and clay sediments (the Prenile deposits and Nile silt units, Figure A.1). In addition, the Upper Oligocene basalt is exposed in the middle (Abu Zaabal Quarries) adjacent to the Miocene sediments of the Hagul Formation (fluviatile sand and gravel, Conoco, 1986). On the other hand, sandy parts in the east belong to the Miocene age (RIGW, 1988).

**Fig. 1:**
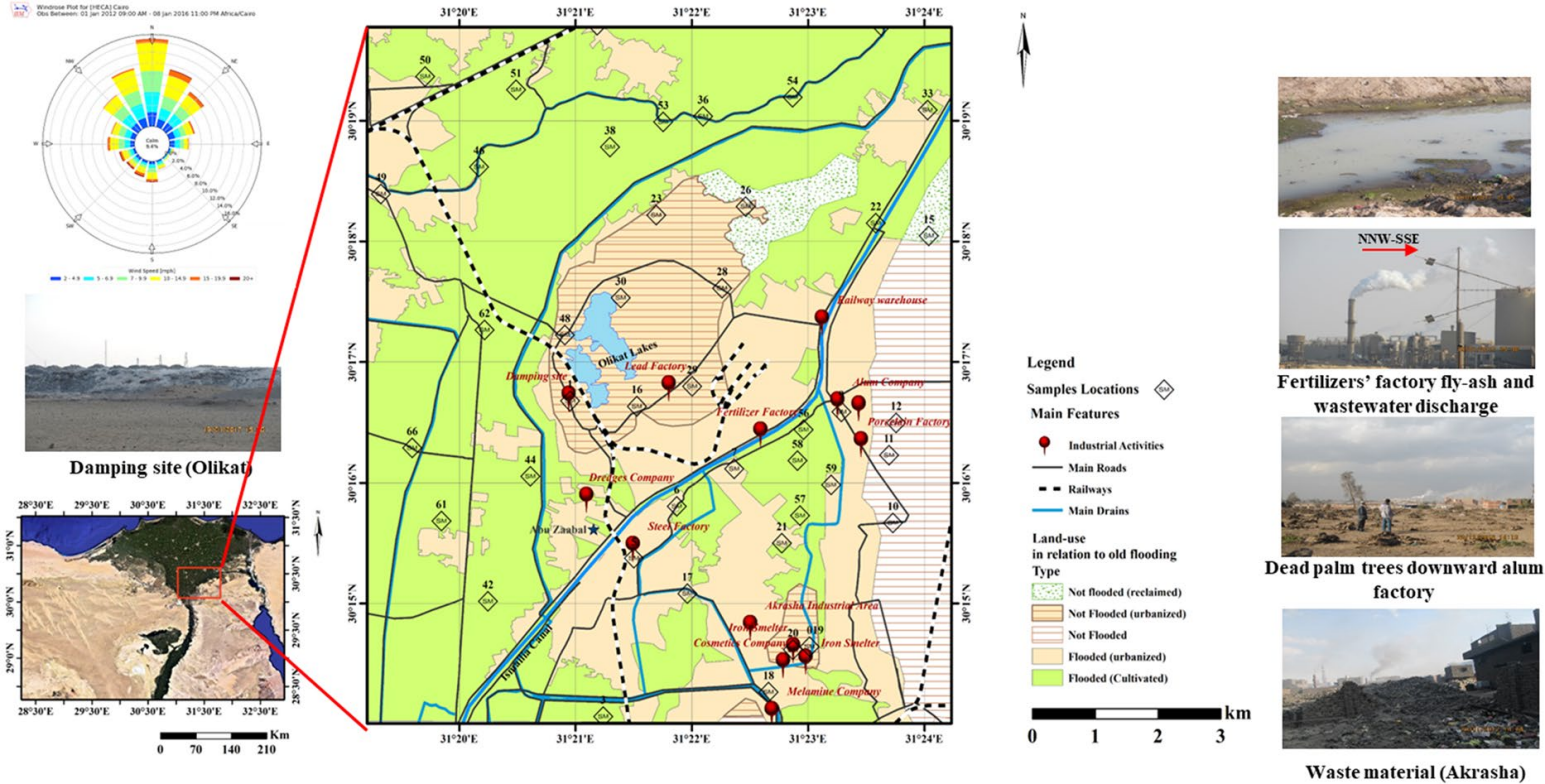
Land use map of the study area with locations of the samples selected for chemical analysis (modified after Guda et al., 2020). The windrose diagram is obtained from IEM where data includes the period from 2012 to 2017)

It was partially submerged during flooding periods before the High dam construction in 1964. This led to spatial and temporal lithological variations (Guda et al., 2020). The flooded parts are mainly cultivated lands with small rural communities, while the non-flooded and reclaimed lands are residential and industrial (Fig. 1). The area is characterized by randomness, where residential, agricultural, and industrial activities greatly overlap. The area comprises different industrial pollution sources: the Abu Zaabal steel factory, two fertilizers factories, a lead smelter, and smaller factories for dredges, porcelain, alum, cosmetics, and plastic industries, in addition to the unsupervised smelting activities in the Akrasha area. The steel factory produced military tools after the 1952 revolution. It was stopped in 2002; however, its pollutive effect is still ongoing as soil renewal has stopped. The well-known Abu Zaabal fertilizers factory is located directly on the Ismailia canal bank, and the other one to the north is still under experimentation. People in the vicinity of the Abu Zaabal fertilizers factory are blaming the factory for respiratory diseases. Atmospherically deposited phosphate salts caused the downwind degradation of cultivated lands. The lead smelter was relocated from Shubra El-Khema to Abu Zaabal since 2000 (Ali et al., 2011). Finally, of great concern is the Akrasha area, which includes several, and not fully known, unsupervised smelting activities. All these activities emit PTEs that spread and are transported by air and water to the soil. Many of them dump solid wastes near the Olikat ponds, which get groundwater out of the Oligocene basalt fractures to fill the depressions created by excessive basalt quarrying activities. These ponds also receive seepage water from the surface and from discharged effluents of the nearby industries.

### Sampling and chemical analysis

Figure 1 subdivides the study area into five categories: flooded, flooded modified by urbanization, never flooded, never flooded modified by reclamation or urbanization considering lithology, and land use. In the winters of 2017 and 2018, we collected seventy-four surface samples from 70 sites covering an area of ca. 80 km^2^. The sampling is described in detail by Guda et al. (2020). Samples were taken from locations with different soil types and land use activities, avoiding wastes and macro-magnetic objects (e.g., wires, iron, debris). Sampling points’ locations were determined using a Garmin GPS instrument. According to Abu El Enain et al. (2010) and the soil map of the Nile delta by Gad and Ali (2011), the collected samples fall within the Estinosol group. North and west are classified under Vetric Torrifluvents, while the east and southern parts are mostly Typic Quartizipsamments and Typic Torripsamments. According to FAO (2006), samples are classified mainly as silty clay, silty loam, and silty loam in agricultural lands (W and N). On the other hand, they are silty loam, loamy sand, and sandy soils in east, middle, and south (Figure A2), which is quietly different from the natural lithofacies distribution (Figure A1) by the effect of ~ 30 years of urbanization/reclamations.

Guda et al. (2020) measured the in situ bulk magnetic susceptibilty (ϰ) in SI units using a MS2D Bartington loop sensor in conjunction with a MS3 probe and Bartsoft software (V. 4.2.1.1). In addition, they subjected all the samples (74 surface samples) to laboratory analysis using an Agico MFK1-FA device at room temperature (at 976 Hz and 200 A/m field), from which mass-specific magnetic susceptibility (x) values were determined. Besides, they conducted physical grain size analysis on 15 surface samples representing all spatial lithological variations in the study area using the Miller and Miller (1987) micro-pipette method. In addition, eight core samples (45 sub-samples) were also subjected to grain size analysis. The results proved the gradation in grain size from fine in the north and west to coarse in the east and south, while grain size distributions with depth indicated a noticeable thinning in the silty layer to the east with downcore coarsening in the sandy fraction.

Forty-six surface samples (Fig. 1) of Guda et al. (2020) were selected for chemical analysis, determining the concentrations of 37 elements by ICP-MS (ACME Lab, Vancouver, Canada). Grinded bulk samples were subjected to digestion in open vessels by heating on a hot plate using a combination of nitric, hydrochloric, and perchloric acids (1:1:1) to dissolve the silicate minerals. A quality check was performed by ACME using reference materials, replicate measurements for samples and blanks to verify the entire analytical process, ensuring the accuracy of calibration solutions. The lowest detection limits for major elements range from 0.01 to 0.001%, while other elements are 0.01–1 mg/kg and 0.5 pbb for Au. The chosen samples express lithological variation, different degrees of pollution, and are well spread over the study area. ArcGIS 10.7 was used for mapping the selected elements.

### Statistical analysis

Basic descriptive statistics, Pearson’s correlation coefficients, and multi-variate analyses were performed using the IBM SPSS software package version 22. To achieve more reliable results, considering lithologic variation, principal component factor analysis (PCA) was carried out on 21 elements measured in a total of 112 samples (the 46 surface samples and 66 core sub-samples of Guda et al. (2020)). The component extraction in this study was carried out using the varimax rotation, with a rule that eigenvalues should be greater than 1.0. The data quality for PCA analysis was tested using the Kaiser–Meyer–Olkin measure of sampling adequacy (KMO test). Finally, cluster analysis was performed to distinguish the dominating effect on the magnetic signal (lithology or pollution).

### Contamination assessment

The enrichment factor (EF) of Reimann and de Caritat (2000) was used to quantify the contaminants’ magnitude relative to a natural (geogenic and/or pedogenic) background. The global average Earth’s crust composition of Rudnick and Gao (2014) is used as a background value to assess the contamination levels of Cu, Zn, Mo, Cd, Sb, Pb, Hg, and As. The used background values are 28, 67, 1.1, 0.09, 0.4, 17, 0.05, and 4.8; respectively. As aluminum is known as a clay proxy, the Al local concentration was used as a reference element to minimize the heterogeneity of the data by the effect of lithological variation. The EF was calculated according to Eq. B.1 (Supplement B). According to Sutherland (2000). This is classified into six enrichment levels: no enrichment (EF < 1), minimal enrichment (1 ≤ EF < 2), moderate enrichment (2 ≤ EF ≤ 5), substantial enrichment (5 ≤ EF ≤ 20), very high enrichment (20 ≤ EF ≤ 40), and extremely high enrichment (EF > 40). Values of EF < 2 indicate that the metal is entirely from crustal materials or natural processes while those > 2 suggest anthropogenic sources.

The overall toxicity level of eight PTEs was assessed by calculating the pollution load index $PLI=\sqrt[n]{CF1xCF2x\dots ..xCFn}$ proposed by Tomlinson et al. (1980), where *n* is the number of elements (here *n* = 8) and *CF* is the contamination factor calculated relative to Rudnick and Gao (2014) Earth’s crust composition (Supplement B, Eq. B.2). A value of PLI = 1 indicates that only baseline levels of pollutants are present, while PLI > 1 would indicate deterioration of the site quality (Hakanson, 1980; Tomlinson et al., 1980).

## Results and discussion

### Geochemical assessment

#### Descriptive statistics and spatial distribution of the studied elements

The measured mean values of elements (Table C.1) are considered relative to Earth’s crust (Rudnick & Gao, 2014; Wedepohl, 1995) and conterminous US soil average compositions (Shacklette & Boerngen, 1984). Ca, P, S, Cu, Zn, As, Cd, Sb, Au, Hg, and Pb show clear enrichments supporting their anthropogenic origin. Fe, Mn, Mg, V, Cr, Co, Ni, Sr, and Mo show enrichments relative to the average soil composition. Of these twenty elements, As, Co, Cr, Cu, Ni, Pb, V, Zn, Ca, Fe, Mn, and P are well-known indicators of anthropogenic processes (Abu Khatita, 2011), and also Cd, Mo, Sb, and Hg are mainly of anthropogenic origin.

**Fig. 2:**
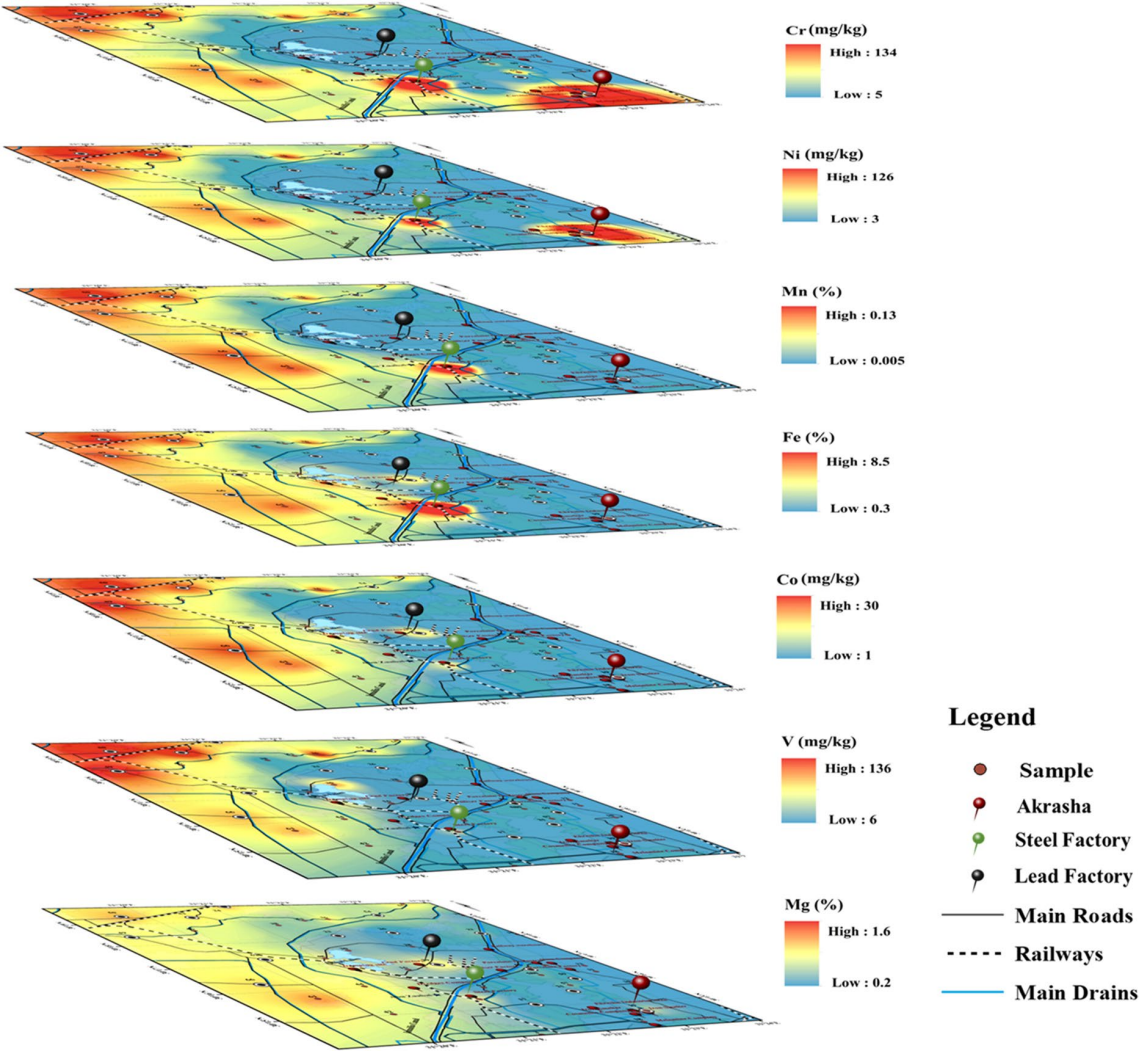
Spatial distribution of Fe, Mn, Mg, V, Co, Ni, and C, related to clayey and/or silt-rich soil

The spatial distributions of Fe, Mn, Mg, V, Co, Ni, and Cr (Fig. 2) are mostly concordant with lithology, with high concentrations in clayey/silty soils in the north and west compromising cultivated lands with small villages and depletion in the sandy central, eastern, and southern parts. This indicates their natural geogenic origin; however, anomalous values of Fe, Mn, Cr, and Ni appear in industrial areas. Maximum concentrations of Fe and Mn (8.5 and 0.13%; respectively) are detected in the steel factory, and Cr and Ni highest concentrations are recorded in Akrasha area (134, 126.1 mg/kg; respectively), followed by the area around the steel factory (109, 69.5 mg/kg; respectively).

**Fig. 3:**
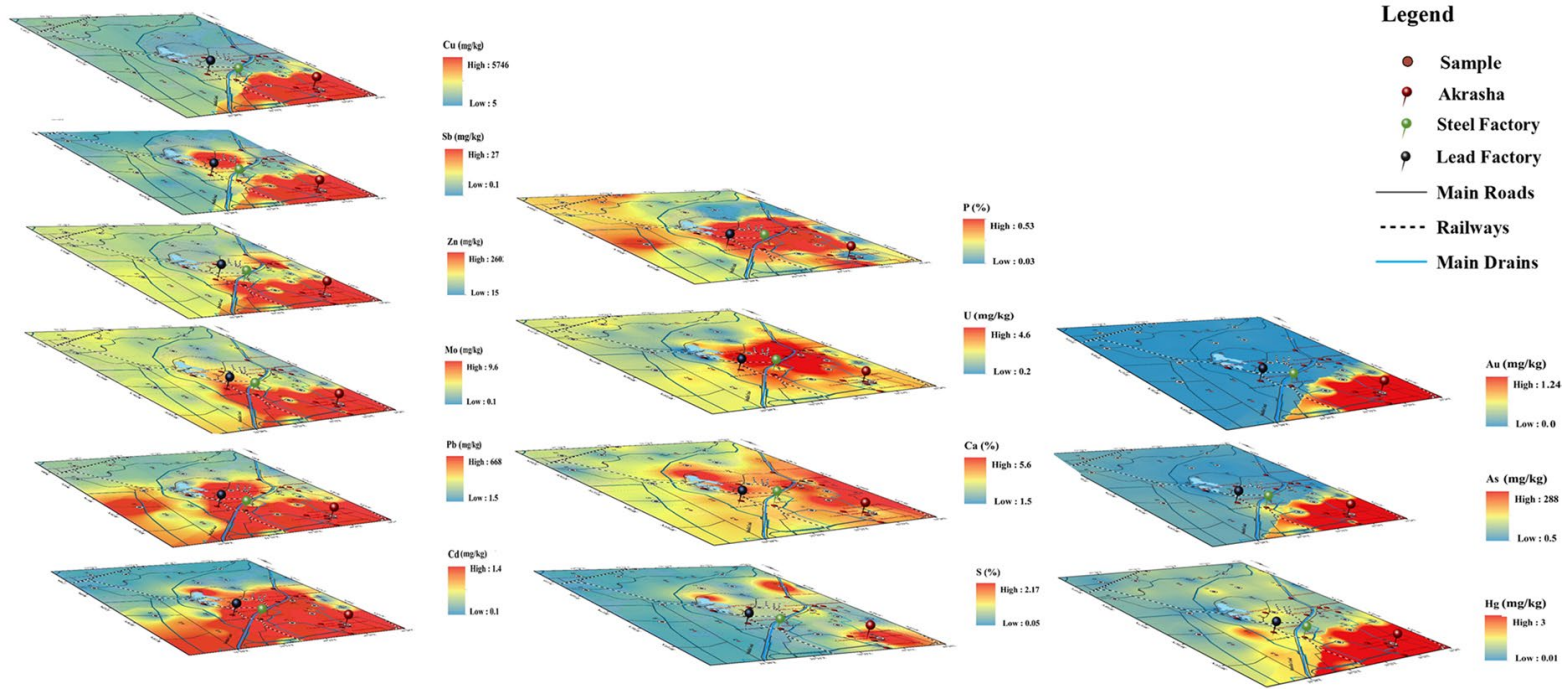
Spatial distribution of anthropogenic related elements; **a**, spatial distribution of Cd, Pb, Mo, Zn, Sb, and Cu, indicative for main industrial activities; **b**, compilation showing the similar concentration distribution of Au, As, and Hg; **c**, spatial distribution of S, Ca, P, and U, characterizing the soil surrounding the Abu Zaabal fertilizers company. The main industrial spots (Akrasha area, steel factory, and lead factory) are marked with red pins

Cd, Pb, Mo, Zn, Sb, Cu, and S distributions are not related to lithology (Fig. 3). Their concentrations are heterogeneous, as reflected in their high standard deviations (Table C.1). The highest concentrations of Cd are found in the steel factory (1.4 mg/kg) and the Akrasha area (0.8–1 mg/kg), and also around phosphate, porcelain, and alum factories, where its concentration is quite high. The Akrasha shows the highest concentrations of Sb, Zn, Cu, Mo, and Pb (26.7, 2602, 5746.2, 9.6, 669.5 mg/kg; respectively). These elements also show high concentrations around the steel factory (1.4, 529, 126.7, 5.7, 366.5 mg/kg; respectively). Besides, high concentrations of Pb and Sb are detected around the lead factory (353.2, 8.7 mg/kg; respectively). The areas near porcelain and alum factories show quite high concentrations of Sb and Zn. No wide distribution of S is found in the study area; however, its concentration is noticeable around the phosphate factory, in the Akrasha area, and in the dumping site near Olikat ponds.

Au, As, and Hg show distinctive distributions (Fig. 3c). Au is only detected in the Akrasha area (12.41 mg/kg), associated with high concentrations of As (288.1 mg/kg) and Hg (2.92 mg/kg). This might be attributed to an unsupervised gold industry. On the other hand, As and Hg have high concentrations in the steel factory area (8.8, 0.11 mg/kg; respectively). Similarly, P and U have closely similar distributions, where P has high values in samples affected by the fertilizers factory (0.525%) and moderately high values in cultivated lands in the north and west where phosphate salts are applied as fertilizers. However, U is only detected in the area receiving dust from the fertilizer factory, which indicates a relationship with the transferred phosphate ore and its removal through processing procedures. Finally, Ca shows higher concentrations in the surroundings of the fertilizers factory to the east, around the dumping site, near the porcelain factory, and near the similar activities (building bricks, etc.) in the Akrasha area.

The Akrasha area, where unsupervised activities prevail, is clearly the main pollution hotspot. High concentrations of most detected pollutants are there. Above all, the noticeably increased values of Au, As, and Hg are striking, from which As is known as the natural pathfinder of Au mineralization (Lestinen et al., 1991; Zhang et al., 2001) and Hg is used in the Au industry in a mixture known as amalgam. The Akrasha area has sandy soil that cannot naturally adsorb the detected high concentrations of Cu, Zn, Sb, Mo, Cd, and Pb, proving their anthropogenic origin.

The high concentrations of Pb, Sb, and Cd in the central part of the study area are attributed to the lead industry. In addition, P, U, and Ca distributions are obviously connected to the Abu Zaabal fertilizers company. Ca is an essential element in phosphate-group minerals, while U is known to be associated with phosphate ores (Fig. 3b). It is worth mentioning that sulfuric acid is the backbone of the fertilizers industry, which produces phosphogypsum as a dump. The main components of phosphogypsum are P, S, and Na. People sometimes use these wastes in cultivation as a secondary fertilizer. However, Ca shows a weak spatial correlation with all elements as it has many different natural and anthropogenic sources in the study area. Finally, the dumping site around Olikat ponds shows significant concentrations of S and Cd as industrial solid wastes from different origins (Fig. 3 b and c).

### Statistical analysis

#### Pearson’s correlation analysis .

Pearson’s correlation results (Table C.2) of lithogenic elements (Fe, Mn, Mg, Co, V, Cr, and Ni) are greatly concordant with their spatial distributions, with moderate to mostly strong correlations (*r* = 0.47–0.94). That assures the dominance of their natural origin in the northern and western parts. Cr and Ni show moderate correlations with Mo, Cd, and Sb (*r* = 0.42–0.67). Cd, Pb, Mo, Zn, Sb, and Cu are well related (*r* = 0.52–0.94), and Pb is moderately correlated with Hg (*r* = 0.46). Exceptionally, Hg has a robust correlation only with Au (*r* = 0.99) and As (*r* = 0.98), which clearly confirms that they are related to the same localized Au-industry in Akrasha. On the other hand, Au and As are weakly to moderately correlated with Pb (0.39 and 0.4; respectively). S is moderately correlated to U (*r* = 0.53) that in turn is strongly correlated to P (*r* = 0.79). Almost all these elements appeared to be spatially concentrated in industrial spots (Fig. 3).

#### Principal component analysis (factor analysis).

Four rotated components representing more than 85% of the total variance were extracted (Table 1). The KMO value is 0.70, indicating sampling adequacy. The first principal component (PC1) explains 36.72% of the total variance. It shows a strong positive association of Fe, Mn, Mg, V, Cr, Co, and Ni, which fits well with their spatial distribution (Fig. 2) and their significant correlations (Table C.2) . In addition, Al, the main clay proxy, has a strong positive coefficient (0.84). Therefore, PC1 is considered to represent natural lithology and agricultural activities. The second component (PC2) accounts for 25.88% of the overall variance and well expressed by Cu, Zn, Mo, Cd, Sb, and Pb, while sulfur has a moderate coefficient (0.43). Almost all these elements have high concentrations in the Akrasha area. Besides, high concentrations of Pb, Sb, and Cd are recorded in the middle part, where the lead factory and the old steel factory are located. In addition, Cd and S showed significant concentrations in the dumping site near Olikat Ponds (Fig. 3 a and b). This indicates the representation of PC2 for most industrial-related heavy metals. The third principal component (PC3) has distinctive high loadings on As, Au, and Hg (0.97–0.99), which obviously indicates the gold industry in the Akrasha area where these elements were detected in extreme concentrations. Finally, PC4 shows high positive coefficients for P, S, and U, which is clearly related to the phosphate industry. The weak correlations between the four components (Table C.3) along with their matching with correlations and spatial distributions reflect the reliability of this analysis in distinguishing the possible sources of the studied elements.

**Table 1:** Rotated component matrix for surface and core samples data (112 samples) with (right) and without (left) introducing susceptibility (the loadings of elements belonging to each component are shown in bold)

	**Component**					**Component**			
	PC1	PC2	PC3	PC4		PC1	PC2	PC3	PC4
**Variance%**	**36.72**	**25.88**	**13.02**	**9.42**	**Variance%**	**36.97**	**24.73**	**12.44**	**9.10**
	**Lithology**	**Industry**				**Lithology**	**Industry**		
					**x**	**0.65**	0.22	− 0.08	0.09
**Al**	**0.84**	0.31	− 0.08	0.12	**Al**	**0.85**	0.23	− 0.04	− 0.01
**Fe**	**0.95**	− 0.04	− 0.03	0.05	**Fe**	**0.95**	− 0.02	− 0.05	0.13
**Mn﻿**	**0.98**	− 0.01	− 0.04	0.06	**Mn**	**0.97**	0.00	− 0.04	0.10
**Mg**	**0.95**	− 0.05	− 0.05	0.12	**Mg**	**0.94**	− 0.07	− 0.04	0.11
**Ca**	− 0.58	0.02	− 0.09	0.31	**Ca**	− 0.57	− 0.01	− 0.08	0.21
**P**	0.27	− 0.03	0.01	**0.81**	**P**	0.24	0.02	− 0.01	**0.89**
**S**	− 0.10	0.43	− 0.06	**0.62**	**S**	− 0.08	0.32	− 0.02	0.36
**V**	**0.97**	− 0.10	− 0.03	0.05	**V**	**0.97**	− 0.10	− 0.03	0.07
**Cr**	**0.84**	0.27	0.00	0.22	**Cr**	**0.84**	0.24	0.01	0.18
**Co**	**0.97**	− 0.12	− 0.04	0.02	**Co**	**0.96**	− 0.12	− 0.04	0.07
**Ni**	**0.93**	0.29	0.04	0.08	**Ni**	**0.94**	0.25	0.05	0.02
**Cu**	− 0.04	**0.85**	0.19	− 0.13	**Cu**	− 0.05	**0.86**	0.18	− 0.10
**Zn**	0.06	**0.94**	0.04	− 0.03	**Zn**	0.06	**0.95**	0.04	− 0.03
**As**	− 0.01	0.13	**0.99**	− 0.02	**As**	− 0.01	0.13	**0.99**	− 0.02
**Mo**	0.20	**0.87**	0.04	0.15	**Mo**	0.23	**0.82**	0.05	0.03
**Cd**	0.02	**0.78**	0.02	0.34	**Cd**	0.03	**0.84**	− 0.01	0.38
**Sb**	0.02	**0.94**	0.06	0.01	**Sb**	0.03	**0.88**	0.08	− 0.11
**Au**	− 0.03	0.11	**0.99**	− 0.02	**Au**	− 0.03	0.11	**0.99**	− 0.02
**Hg**	− 0.05	0.21	**0.97**	0.01	**Hg**	− 0.05	0.21	**0.97**	0.00
**Pb**	0.04	**0.84**	0.34	0.04	**Pb**	0.04	**0.88**	0.32	0.09
**U**	0.08	0.01	0.00	**0.95**	**U**	0.07	0.02	− 0.01	**0.92**

### Contamination assessment

In order to assess the potential contamination severity of the studied pollutants, EF and PLI were calculated for PC2 (Cu, Zn, Mo, Cd, Sb, and Pb), as well as Hg and As from PC3. These PTEs were identified through mapping, statistics, and factor analysis as highly toxic anthropogenic-related elements.

### Enrichment factor

According to mean EF values, elements are arranged as follows: Cu > Pb > Hg > Sb > Cd > Zn > As > Mo (Table C.4). They mostly show exceptional enrichments in the Akrasha area. Mo and Cd have their highest enrichments around the steel factory (70.83, 23.59; respectively). Cd is highly enriched around the fertilizer factory (59.58; sample 7, Table C.5) and to the southeast (alum and porcelain factories) and has moderate to substantial enrichments near the lead factory (sample 29) and the dumping site. Generally, the southern part of the area has high and very high contamination levels of Pb and Cu, as well as a substantial contamination with Sb. On the other hand, the steel factory is highly to very highly contaminated with Cu, Zn, Cu, and Pb (98.16) and substantially contaminated with As (8.35), while the lead factory is affected by very high contamination of Pb (129.22) and Sb (135.31). The rest of the area has no to moderate enrichments of these pollutants except for the porcelain factory where very high contamination with Zn is detected (35.95; sample 9).

#### Pollution load index

**Fig. 4:**
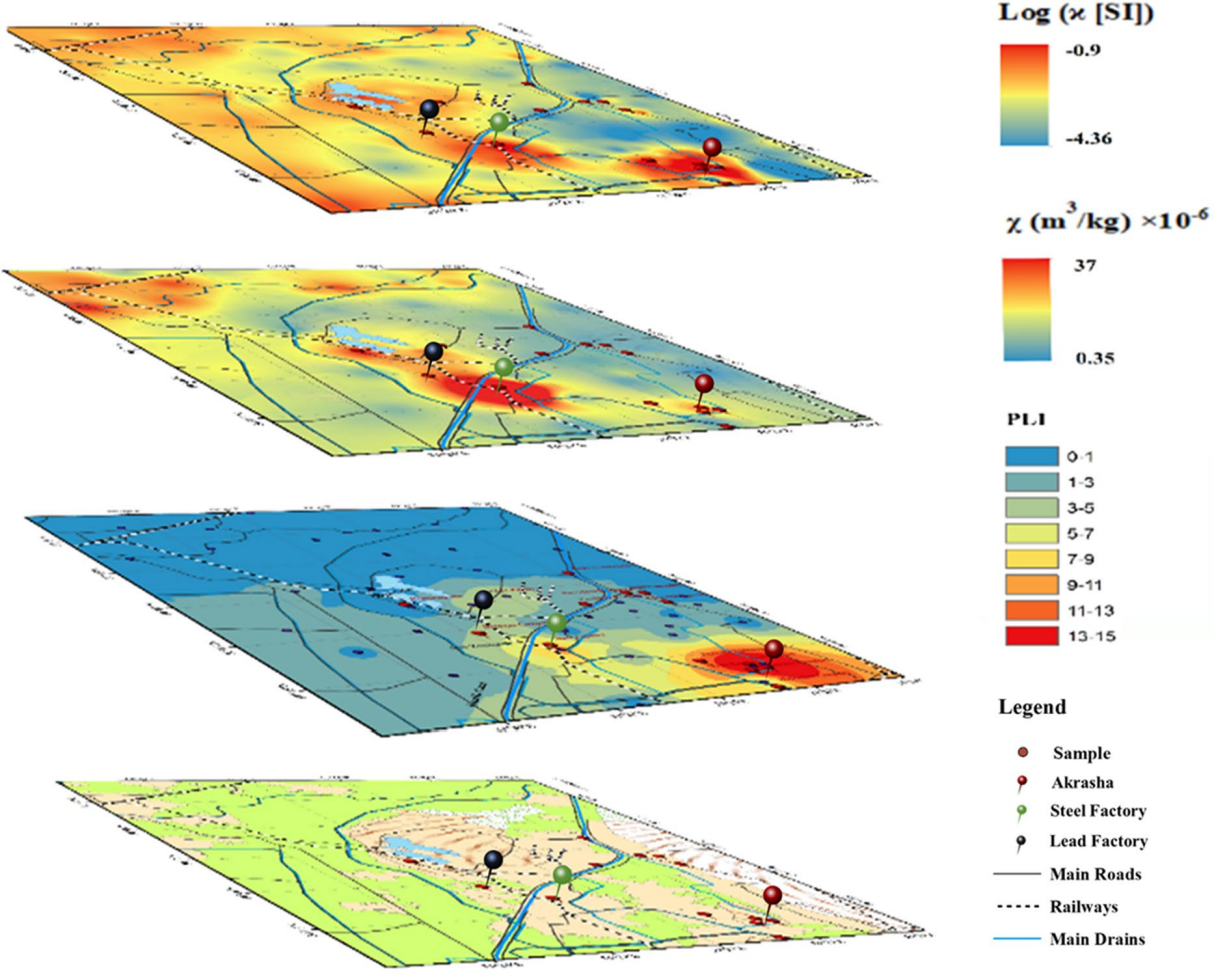
Spatial distribution of in situ bulk susceptibility (ϰ) in logarithmic scale and mass-specific susceptibility ($x$) based on topsoil samples (after Guda et al., 2020), in comparison to the pollution load index (PLI) and the current land use of the study area

The pollution load index for surface samples (Fig. 4) ranges between 0.21 and 15.84, with a median of ~ 0.8. This indicates that the pollution level in the study area ranges from no pollution (PLI < 1) to extremely polluted. The highest PLI is found in the Akrasha area (15.84), followed by the areas of the steel factory (PLI 5.53), the lead factory (PLI 2.34), and the fertilizers factory (PLI 1.12).

### Chemistry and contamination indices in relation to magnetic susceptibility

The area’s complexity is greatly reflected in the magnetic signal. To be able to use magnetic proxy in PTE pollution screening, we need to understand how it could be translated in terms of geochemistry data. A monitoring plan could be established later based on these relations, along with the spatial distribution of lithology and polluted spots. Pollutants are mainly distributed in the southern and middle parts where the main hotspots are located, i.e., the Akrasha area, the steel factory, and the lead factory (Figs. 3 and 4). This corresponds with Guda et al. (2020), who found the highest $x$*-*values in the south and middle. Except for the area surrounding the steel factory, these parts are mostly sandy, either naturally or by urbanization-related activities, and the magnetic signal is primarily derived from anthropogenic sources. However, high $x$-values in the northern and western parts with limited pollution are attributed to silty and/or clayey soil (Guda et al., 2020), which reveals a correspondence with lithogenic elements distribution (Fig. 2). According to these observations, magnetic susceptibility can reflect pollution in industrial areas in the middle and southern sandy parts; however, areas where the soil has significant magnetic susceptibility values should be cautiously handled.

#### Correlations of magnetic susceptibility and heavy metals

The numerical correlations between *x* and PTE concentrations (Table B.2) and PLI with such interference of anthropogenic contaminations and soil properties are complex. Despite that *x* shows a clear spatial correspondence with the industrial spots (Fig. 4), it is weakly correlated with the PLI (*r* = 0.17). Only Cd and Mo are moderately correlated with *x* (*r* of 0.54 and 0.42; respectively). On the other hand, *x* is moderately to strongly correlated with lithogenic elements (Fe, Mn, Mg, V, Cr, Co, and Ni) in surface samples. In order to resolve this relation complexity, we repeated PCA by introducing *x* to the analyzed factors (Table 2). The KMO test is significant (*P*-value < 0) and the KMO value is 0.70, indicating sample number adequacy. *x* is well represented with lithogenic component by 65% (Table 1), indicating their great dependence on the lithogenic element distribution.

**Table 2:** Pearson’s correlation coefficient between mass specific susceptibility ($x$), heavy metal concentrations, and other studied risk indices in the two clusters

	$x$	**Cu**		**Zn**	**Mo**	**Cd**	**Sb**	**Pb**	**Hg**	**As**	**PLI**
Cluster 1 (22 samples)											
$x$	1.00		0.34	0.01	0.14	− 0.30	0.10	− 0.06	− 0.42	− 0.22	− 0.11
Sig			0.14	0.96	0.55	0.20	0.69	0.79	0.07	0.36	0.63
Cu	0.34		1.00	0.73	0.09	− 0.46	0.19	0.29	− 0.07	0.56	0.36
Sig	0.14			0.00	0.69	0.04	0.43	0.21	0.78	0.01	0.12
Zn	0.01		0.73	1.00	0.08	− 0.16	0.33	0.60	0.07	0.77	0.64
Sig	0.96		0.00		0.72	0.51	0.15	0.01	0.78	0.00	0.00
Mo	0.14		0.09	0.08	1.00	0.28	0.48	0.24	0.09	− 0.02	0.40
Sig	0.55		0.69	0.72		0.23	0.03	0.31	0.70	0.95	0.08
Cd	− 0.30		− 0.46	− 0.16	0.28	1.00	0.23	0.11	0.21	0.01	0.26
Sig	0.20		0.04	0.51	0.23		0.32	0.65	0.38	0.97	0.26
Sb	0.10		0.19	0.33	0.48	0.23	1.00	0.839^**^	0.20	0.17	0.87
Sig	0.69		0.43	0.15	0.03	0.32		0.00	0.41	0.47	0.00
Pb	− 0.06		0.29	0.60	0.24	0.11	0.84	1.00	0.09	0.34	0.85
Sig	0.79		0.21	0.01	0.31	0.65	0.00		0.72	0.14	0.00
Hg	− 0.42		− 0.07	0.07	0.09	0.21	0.20	0.09	1.00	0.17	0.44
Sig	0.07		0.78	0.78	0.70	0.38	0.41	0.72		0.48	0.05
As	− 0.22		0.56	0.77	− 0.02	0.01	0.17	0.34	0.17	1.00	0.53
Sig	0.36		0.01	0.00	0.95	0.97	0.47	0.14	0.48		0.02
PLI	− 0.11		0.36	0.64	0.40	0.26	0.87	0.85	0.44	0.53	1.00
Sig	0.63		0.12	0.00	0.08	0.26	0.00	0.00	0.05	0.02	
Cluster 2 (24 samples)											
$x$	1.00		0.85	0.84	0.56	0.68	0.72	0.77	0.10	0.07	0.65
Sig			0.00	0.00	0.00	0.00	0.00	0.00	0.65	0.75	0.00
Cu	**0.85**		1.00	0.95	0.58	0.78	0.79	0.93	0.28	0.24	0.81
Sig	0.00			0.00	0.00	0.00	0.00	0.00	0.18	0.25	0.00
Zn	**0.84**		0.95	1.00	0.70	0.87	0.87	0.90	0.15	0.10	0.79
Sig	0.00		0.00		0.00	0.00	0.00	0.00	0.47	0.65	0.00
Mo	**0.56**		0.58	0.70	1.00	0.78	0.96	0.65	0.20	0.14	0.77
Sig	0.00		0.00	0.00		0.00	0.00	0.00	0.35	0.51	0.00
Cd	**0.68**		0.78	0.87	0.78	1.00	0.86	0.78	0.11	0.06	0.73
Sig	0.00		0.00	0.00	0.00		0.00	0.00	0.59	0.79	0.00
** Sb**	**0.72**		0.79	0.87	0.96	0.86	1.00	0.81	0.20	0.14	0.85
Sig	0.00		0.00	0.00	0.00	0.00		0.00	0.34	0.51	0.00
** Pb**	**0.77**		0.93	0.90	0.65	0.78	0.81	1.00	0.51	0.45	0.92
Sig	0.00		0.00	0.00	0.00	0.00	0.00		0.01	0.03	0.00
** Hg**	0.10		0.28	0.15	0.20	0.11	0.20	0.51	1.00	0.99	0.68
Sig	0.65		0.18	0.47	0.35	0.59	0.34	0.01		0.00	0.00
** As**	0.07		0.24	0.10	0.14	0.06	0.14	0.45	0.99	1.00	0.63
Sig	0.75		0.25	0.65	0.51	0.79	0.51	0.03	0.00		0.00
** PLI**	**0.65**		0.81	0.79	0.77	0.73	0.85	0.92	0.68	0.63	1.00
Sig	0.00		0.00	0.00	0.00	0.00	0.00	0.00	0.00	0.00	

#### Cluster analysis

As previously discussed, the magnetic susceptibility distribution is affected by lithogenic and PTE distributions. So, we aimed to separate samples affected by lithologic elements. In this way, the relationship between *x* and PTE concentrations and their contamination levels could be more clearly inspected. For this purpose, we conducted a two-step cluster analysis to classify samples based on lithogenic element concentrations. The model classified the 46 samples into a 22-sample cluster (Cl-1) including samples rich in lithogenic elements and a second cluster (Cl-2) including 24 samples depleted in such elements (Fig. C.1). The average silhouette measure of cohesion inside the clusters is measured by 0.70, reflecting a good cluster quality and homogeneity in each cluster composition, while the ratio between the Cl-2 and Cl-1 is 1.09 reflecting an acceptable clustering quality. The cluster membership of each sample is shown in Table C.4. In order to understand how *x* is represented in each cluster, its relation to HMs in each cluster was performed separately.

##### Cluster 1.

Cl-1 is enriched with lithogenic elements (Fig. C.1). Samples are mostly silt and/or clay and stem from cultivated parts. Basalt and basalt-rich samples near the basalt quarry in the middle are also included. The area represented by Cl-1 (N, NW, W, and the middle parts) is relatively depleted in the concerned PTEs. Cl-1 includes Olikat ponds, the steel factory, the lead factory, and the dumping site as well. The correlation matrix was calculated after excluding sample no. 5 (steel factory), which is clayey with anomalous PTE concentrations, to avoid misleading correlations by outlier effect. Unlike lithogenic elements, PTEs are weakly related to magnetic susceptibility (Table 2). This proves that the susceptibility values in the represented area are mainly reflecting the primary soil composition rather than pollution.

##### Cluster 2.

Cl-2 has lower lithogenic elements’ concentrations (Fig. C.1). On the other hand, they are rich in PTEs compared to Cl-1 (Fig. C.2). Samples represent sandy soil in the east, southeast, and south. This cluster includes the Akrasha area and its surroundings, the Abu Zaabal fertilizer factory, the porcelain factory, the alum factory, etc. These samples mainly represent downwind soils affected by industrial activities and receive most of the emitted fly ash. The correlation between the studied PTEs and *x* (Table 2) revealed that Cu, Zn, Cd, Sb, and Pb have strong correlations with *x* (*r* = 0.68–0.85) and moderate correlation with Mo (*r* = 0.56). The weak correlations found with Hg and As (r of 0.1 and 0.07; respectively) are attributed to their localized distribution in the Akrasha area. In addition, *x* shows a good correlation with PLI (*r* = 0.65), rather than the very weak correlation (*r* = 0.17) calculated without clustering.

PTE mean concentrations of the two clusters (Fig. C.2) showed the clear enrichment of Cl-2 with PTEs compared to Cl-1. This supports the hypothesis that lithogenic elements in the west and northwest (represented in Cl-1) are responsible for high pedogenic *x* values, while in industrial areas (represented by Cl-2), *x* is attained to PTE concentrations which proves its anthropogenic origin.

## Conclusions

- The effectiveness of magnetic proxies to recognize anthropogenic signals in areas with complex interfering settings such as the current study area is poorly investigated so far. To a great extent, magnetic susceptibility is affected by lithology and grain size variation which limits its efficiency in expressing pollution. Despite the spatial correspondence of $x$ to PTEs and PLI distributions, their numerical correlations were not expressive. The overall numerical correlation of $x$ with PTEs is weak to intermediate, while it is moderate to strong with lithogenic elements. However, comparing lithogenic elements and PTE concentrations along with *x-*values in each cluster supports the correspondence of lithology with elevated *x*-values in silt and/or clay-rich soil samples as well as PTE concentrations in industrial sandy soils. Correspondence between magnetic maps and chemistry data with land use, especially in industrial areas and the downwind affected area, reflects the potential of magnetic proxy methods for qualitative PTE pollution pre-delineation of the polluted spots, provided that lithological conditions are carefully considered.
- Potentially toxic elements (Cd, Pb, Mo, Zn, Sb, Cu, Hg, As) show the highest contamination levels in the Akrasha area (PLI = 15.4), the steel factory (PLI = 5.53), and the lead factory (PLI = 2.34).
- PCA and cluster analysis are found to be very helpful in the visualization and construction of reliable and effective relations in this study area with a strong heterogenic nature. Two clusters can be distinguished, one rich in lithogenic elements and including silt and clay-rich samples with lower concentrations of HMs, the other depleted in lithogenic elements with higher concentrations of PTEs, and mostly sandy samples. Mapping and PCA categorize the studied elements into four groups; a lithogenic (N, NW, and W) and three anthropogenic ones (HMs near industrial spots, Au industry Akrasha area, and P fertilizers industry).
- Statistical tools were so helpful to unravel ambiguities and discriminate between the different effects in the current study area. It is highly recommended to test such type of studies in areas with similar conditions in Egypt and other Middle East developing countries.

### Supplementary Information

Below is the link to the electronic supplementary material.

Supplementary file1 (DOCX 709 KB)

## Data Availability

The data is available upon request.
