# Supplementary material for: Integrated geochemical and magnetic potentially toxic elements assessment: a statistical solution discriminating anthropogenic and lithogenic magnetic signals in a complex area of the southeast Nile Delta
Source: Environ Monit Assess. 2024 Feb 16;196(3):272. doi: 10.1007/s10661-024-12408-5 (PMC10873446; doi:10.1007/s10661-024-12408-5)
Supplement: Supplementary file 1 — Supplementary file1 (DOCX 709 KB) [file 10661_2024_12408_MOESM1_ESM.docx]

Appendices for

**Integrated geochemical and magnetic potentially toxic elements assessment: A statistical solution discriminating anthropogenic and lithogenic magnetic signals in a complex area of the southeast Nile delta**

Alshymaa Mohammad Guda^a*^, Ahmed Mohamed El Kammar^b^, Hend Said Abu Salem ^b^, Atef Mohammady Abu Khatita^c,d^, Mohamed Abdelwahed Mohamed ^b^, Ibrahim Aly El-Hemaly^a^, Esmat Mohamed Abd Elaal^a^, Hatem Hamdy Odah^a^, Erwin Appel^e^

_a Earth’s Geomagnetism lab, National Research Institute of Astronomy and Geophysics (NRIAG), P.box:11421, Helwan, Egypt_

_b Geology Department, Faculty of Sciences, Cairo University, Giza, Egypt_

_c Geology Department, Faculty of sciences, Al-Azhar University, Cairo, Egypt_

_d Geology Department, College of Science, Taibah University, Taibah, Saudi Arabia_

_e Geosciences Department, Tübingen University, Tübingen, Germany_

E-mail address: [shimaaguda@yahoo.com](mailto:shimaaguda@yahoo.com), [alshymaa@nriag.sci.eg](mailto:alshymaa@nriag.sci.eg)

**Appendices**

**Appendix A: Lithology and soil classification of the study area**

**
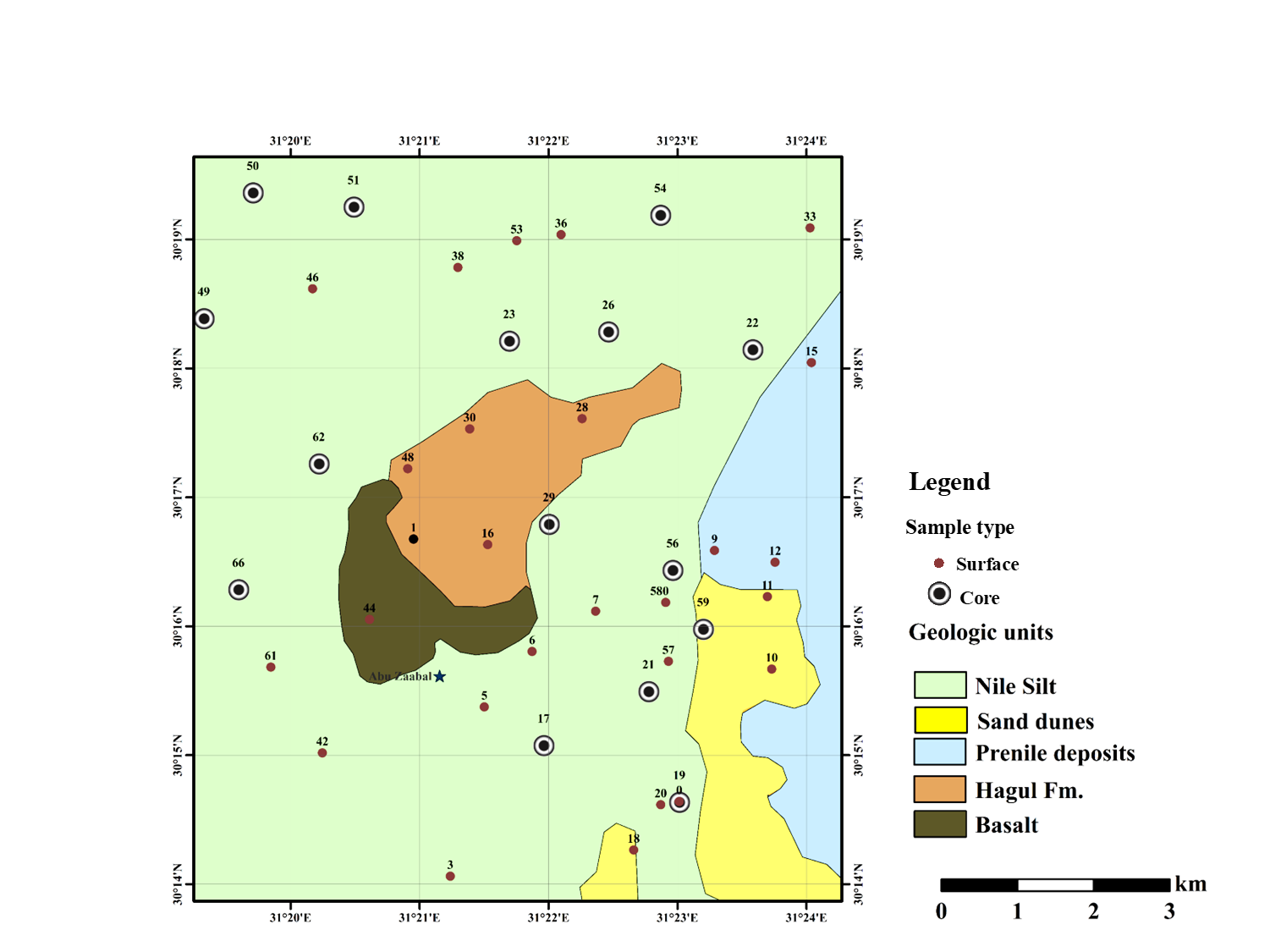
**

**Figure A1:** Lithologic map of the study area (After Conoco 1986) with sampling locations.


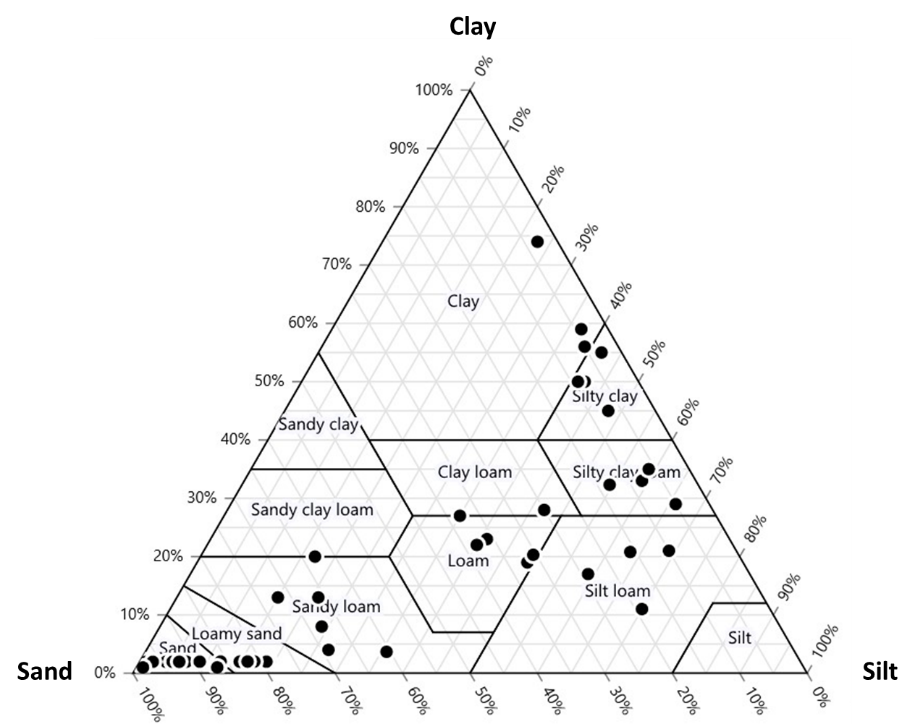


**Figure A.2:** Soil samples classification (According to FAO 2006)

**Appendix B: Contamination risk assessment calculations:**

**B.1. Enrichment factor (EF)**

The enrichment factor for the elements those are identified to have a dangerous impact on human health and related to anthropogenic activities is calculated using the following equation (**Jadoon et al., 2018**):

$EF=(\frac{Cx}{Cr})sample/(\frac{Cx}{Cr}) background$ (Eq. B.1)

where, $Cx$ is the assessed element concentration, and $Cr$ is the reference element concentration which is Al here. The background concentrations were chosen to be that of the Earth’s crust composition according to **Rudnick** **and Gao (2014).** According to **Sutherland (2000)**, EF is classified into six enrichment levels: no enrichment (EF < 1), minimal enrichment (1≤ EF < 2), moderate enrichment (2 ≤ EF ≤ 5), substantial enrichment (5 ≤ EF ≤ 20), very high enrichment (20 ≤ EF ≤ 40), and extremely high enrichment (EF > 40). The EF values lower than 2 indicate that the metal is entirely from crustal materials or natural processes while higher values suggest anthropogenic sources.

**B. 2. Contamination factor**

CF is calculated according to the following equation:

$Cf=C(element)/C(background)$ (Eq. B.2)

where, C element is the element concentration measured in the sample and C background is the background value of that element. The background used in this study is the average Earth’s crust composition of **Rudnick and Gao (2014**). The CF index has four categories to describe the element contamination as follows; CF < 1 indicates low contamination, 1 ≤ CF < 3 refers to the moderate contamination, 3 ≤ CF < 6 refers considerable contamination, and CF>6 indicates a very high contamination. The geo-accumulation index (I_geo_) is defined by **Müller (1969)** to assess the enrichment degree of the contamination by an element taking into consideration the geochemical background values, the anthropogenic pollution, and the effect of natural diagenesis. It depends on comparing the element concentration by the original preindustrial global concentration in the sediments.

**Appendix C: Results**

**Table C.1: Comparison between the mean concentration of the studied elements and the average Earth’s crust and soil composition**

| **Element** | **Unit** | **Min.** | **Max.** | **Mean** | **S. D.** | **Mean** | **Enrichment ratio (with Al reference)** | | |
| --- | --- | --- | --- | --- | --- | --- | --- | --- | --- |
|  |  |  |  |  |  |  | **Rudnick &**  **Gao 2014** | **Wedepohl**  **1995** | **Shacklette &** **Boerngen**  **1984** |
| ***Ti*** | **%** | 0.01 | 0.28 | 0.11 | 0.07 | 0.11 | 1.24 | 1.16 | 1.14 |
| ***Al*** | **%** | 0.19 | 10.00 | 1.84 | 1.54 | 1.84 | 1.00 | 1.00 | 1.00 |
| ***Fe*** | **%** | 0.28 | 8.53 | 2.67 | 1.73 | 2.67 | 3.01 | 2.67 | 3.78 |
| ***Mn*** | **mg/kg** | 52 | 1300 | 485.87 | 300 | 485.87 | 2.69 | 2.94 | 3.76 |
| ***Mg*** | **%** | 0.18 | 1.62 | 0.71 | 0.36 | 0.71 | 2.12 | 1.41 | 4.15 |
| ***Ca*** | **%** | 1.52 | 5.61 | 2.90 | 1.02 | 2.90 | **5.01** | **3.26** | **8.05** |
| ***Na*** | **%** | 0.02 | 1.61 | 0.22 | 0.32 | 0.22 | 0.40 | 0.40 | 0.95 |
| ***K*** | **%** | 0.05 | 0.82 | 0.23 | 0.15 | 0.23 | 0.43 | 0.46 | 0.39 |
| ***P*** | **%** | 0.03 | 0.53 | 0.13 | 0.09 | 0.13 | **8.59** | **7.26** | **12.47** |
| ***S*** | **%** | 0.05 | 2.17 | 0.28 | 0.48 | 0.28 | **19.88** | **17.30** | **5.93** |
| ***Sc*** | **mg/kg** | 0.60 | 13.20 | 5.48 | 3.74 | 5.48 | 1.73 | 1.48 | 1.87 |
| ***V*** | **mg/kg** | 6.00 | 136.00 | 64.59 | 36.81 | 64.59 | 2.95 | 2.85 | 2.84 |
| ***Cr*** | **mg/kg** | 5.00 | 134.00 | 43.43 | 27.94 | 43.43 | 2.09 | 1.38 | 3.00 |
| ***Co*** | **mg/kg** | 1.30 | 29.60 | 13.85 | 8.52 | 13.85 | 3.55 | 2.50 | 5.28 |
| ***Ni*** | **mg/kg** | 2.70 | 126.10 | 33.20 | 22.81 | 33.20 | 3.13 | 2.56 | 6.52 |
| ***Cu*** | **mg/kg** | 4.90 | 5746.20 | 252.40 | 893.01 | 252.40 | **39.93** | **43.68** | **37.92** |
| ***Zn*** | **mg/kg** | 15.00 | 2602.00 | 184.00 | 411.78 | 184.00 | **12.16** | **12.25** | **9.79** |
| ***Ga*** | **mg/kg** | 1.00 | 16.00 | 5.28 | 3.42 | 5.28 | 1.34 | 1.52 | 1.04 |
| ***As*** | **mg/kg** | 0.50 | 288.10 | 7.98 | 42.25 | 7.98 | **7.37** | **20.31** | **3.92** |
| ***Sr*** | **mg/kg** | 62.00 | 416.00 | 132.80 | 68.76 | 132.80 | 1.84 | 1.73 | 2.83 |
| ***Mo*** | **mg/kg** | 0.10 | 9.60 | 0.84 | 1.66 | 0.84 | 3.40 | 3.32 | 3.59 |
| ***Cd*** | **mg/kg** | 0.10 | 1.40 | 0.22 | 0.25 | 0.22 | **11.02** | **9.69** | 2.86 |
| ***Sb*** | **mg/kg** | 0.10 | 26.70 | 1.71 | 5.20 | 1.71 | **18.92** | **24.64** | 9.09 |
| ***Ba*** | **mg/kg** | 21.00 | 406.00 | 120.46 | 80.83 | 120.46 | 0.86 | 0.89 | 0.70 |
| ***La*** | **mg/kg** | 3.00 | 25.00 | 13.46 | 6.53 | 13.46 | 1.92 | 1.94 | 1.15 |
| ***Au*** | **μg/kg** | 0.5 | 12410 | 293.02 | 1830 | 293.02 | **884.50** | **507.06** | - |
| ***Hg*** | **mg/kg** | 0.01 | 2.92 | 0.14 | 0.43 | 0.14 | **12.09** | **14.77** | **6.01** |
| ***Pb*** | **mg/kg** | 1.50 | 669.50 | 69.58 | 134.20 | 69.58 | **18.13** | **20.34** | **11.11** |
| ***Th*** | **mg/kg** | 0.10 | 3.80 | 1.66 | 0.97 | 1.66 | 0.70 | 0.84 | 0.49 |
| ***U*** | **mg/kg** | 0.20 | 4.60 | 0.83 | 0.83 | 0.83 | 1.36 | 2.11 | 0.92 |

**Table C.2*:*** *Correlation coefficients between the studied elements and magnetic susceptibility.*

|  | ***Χ*** | ***Fe*** | ***Mn*** | ***Mg*** | ***Ca*** | ***P*** | ***S*** | ***V*** | ***Cr*** | ***Co*** | ***Ni*** | ***Cu*** | ***Zn*** | ***As*** | ***Mo*** | ***Cd*** | ***Sb*** | ***Au*** | ***Hg*** | ***Pb*** | ***U*** |
| --- | --- | --- | --- | --- | --- | --- | --- | --- | --- | --- | --- | --- | --- | --- | --- | --- | --- | --- | --- | --- | --- |
| ***χ*** | 1.00 |  |  |  |  |  |  |  |  |  |  |  |  |  |  |  |  |  |  |  |  |
| **Fe** | **0.76** | 1.00 |  |  |  |  |  |  |  |  |  |  |  |  |  |  |  |  |  |  |  |
| **Mn** | **0.67** | **0.94** | 1.00 |  |  |  |  |  |  |  |  |  |  |  |  |  |  |  |  |  |  |
| **Mg** | 0.34 | **0.81** | **0.86** | 1.00 |  |  |  |  |  |  |  |  |  |  |  |  |  |  |  |  |  |
| **Ca** | -0.31 | -0.37 | -0.39 | -0.29 | 1.00 |  |  |  |  |  |  |  |  |  |  |  |  |  |  |  |  |
| **P** | -0.08 | -0.04 | -0.07 | -0.05 | 0.13 | 1.00 |  |  |  |  |  |  |  |  |  |  |  |  |  |  |  |
| **S** | -0.15 | -0.22 | -0.16 | 0.04 | 0.38 | 0.12 | 1.00 |  |  |  |  |  |  |  |  |  |  |  |  |  |  |
| **V** | **0.48** | **0.87** | **0.90** | **0.87** | -0.39 | -0.05 | -0.15 | 1.00 |  |  |  |  |  |  |  |  |  |  |  |  |  |
| **Cr** | **0.43** | **0.47** | **0.61** | **0.47** | -0.24 | 0.03 | 0.25 | **0.47** | 1.00 |  |  |  |  |  |  |  |  |  |  |  |  |
| **Co** | **0.48** | **0.91** | **0.91** | **0.92** | -0.44 | -0.03 | -0.23 | **0.95** | 0.40 | 1.00 |  |  |  |  |  |  |  |  |  |  |  |
| **Ni** | **0.48** | **0.59** | **0.72** | **0.68** | -0.40 | -0.16 | 0.23 | **0.62** | **0.81** | **0.59** | 1.00 |  |  |  |  |  |  |  |  |  |  |
| **Cu** | 0.09 | -0.21 | -0.13 | -0.22 | -0.03 | -0.21 | 0.17 | -0.24 | 0.07 | -0.27 | 0.21 | 1.00 |  |  |  |  |  |  |  |  |  |
| **Zn** | 0.20 | -0.12 | -0.03 | -0.16 | -0.06 | -0.18 | 0.26 | -0.20 | 0.24 | -0.22 | 0.34 | **0.94** | 1.00 |  |  |  |  |  |  |  |  |
| **As** | -0.04 | -0.12 | -0.13 | -0.15 | -0.11 | -0.15 | -0.01 | -0.13 | -0.03 | -0.16 | 0.09 | 0.26 | 0.12 | 1.00 |  |  |  |  |  |  |  |
| **Mo** | **0.41** | 0.09 | 0.14 | 0.04 | -0.06 | -0.13 | **0.50** | -0.06 | **0.57** | -0.11 | **0.67** | **0.52** | **0.69** | 0.15 | 1.00 |  |  |  |  |  |  |
| **Cd** | **0.54** | 0.13 | 0.11 | -0.15 | 0.08 | 0.16 | 0.30 | -0.17 | **0.42** | -0.19 | 0.34 | **0.53** | **0.69** | 0.07 | **0.79** | 1.00 |  |  |  |  |  |
| **Sb** | 0.06 | -0.18 | -0.09 | -0.07 | -0.05 | -0.17 | **0.46** | -0.19 | 0.31 | -0.22 | **0.50** | **0.77** | **0.84** | 0.16 | **0.83** | **0.58** | 1.00 |  |  |  |  |
| **Au** | -0.06 | -0.14 | -0.15 | -0.17 | -0.10 | -0.14 | -0.02 | -0.15 | -0.04 | -0.17 | 0.06 | 0.26 | 0.11 | **1.00** | 0.12 | 0.05 | 0.15 | 1.00 |  |  |  |
| **Hg** | -0.07 | -0.18 | -0.18 | -0.20 | -0.09 | -0.14 | 0.02 | -0.19 | 0.03 | -0.22 | 0.09 | 0.31 | 0.18 | **0.98** | 0.20 | 0.12 | 0.23 | **0.99** | 1.00 |  |  |
| **Pb** | 0.32 | 0.02 | 0.01 | -0.13 | -0.04 | -0.12 | 0.16 | -0.18 | 0.24 | -0.16 | 0.31 | **0.80** | **0.82** | 0.40 | **0.66** | **0.72** | **0.77** | 0.39 | **0.46** | 1.00 |  |
| **U** | -0.14 | -0.13 | -0.14 | -0.06 | 0.37 | **0.79** | **0.53** | -0.14 | 0.06 | -0.16 | -0.12 | -0.12 | -0.09 | -0.07 | 0.01 | 0.24 | -0.07 | -0.08 | -0.08 | -0.08 | 1 |

**Table C.3:** *The extracted components’ transformation matrix before (left) and after (right) introducing ꭓ.*

| **Component Transformation Matrix** | | | | |  | **Component Transformation Matrix** | | | | |
| --- | --- | --- | --- | --- | --- | --- | --- | --- | --- | --- |
| **Component** | **PC1** | **PC2** | **PC3** | **PC4** |  | **Component** | **PC1** | **PC2** | **PC3** | **PC4** |
| **PC1** | 0.95 | 0.28 | 0.01 | 0.16 |  | **PC1** | 0.95 | 0.26 | 0.00 | 0.15 |
| **PC2** | -0.28 | 0.87 | 0.39 | 0.11 |  | **PC2** | -0.25 | 0.86 | 0.39 | 0.05 |
| **PC3** | 0.14 | -0.29 | 0.86 | -0.39 |  | **PC3** | 0.13 | -0.27 | 0.86 | -0.32 |
| **PC4** | -0.08 | -0.28 | 0.33 | 0.90 |  | **PC4** | -0.07 | -0.28 | 0.33 | 0.85 |

**Table C.4:** *The calculated risk factors for the studied samples based on background concentrations according to Rudnick and Gao (2014).*

| **Enrichment factor** | | | | | | | | |
| --- | --- | --- | --- | --- | --- | --- | --- | --- |
|  | **Cu** | **Zn** | **Mo** | **Cd** | **Sb** | **Pb** | **Hg** | **As** |
| **Mean** | 54.38 | 15.70 | 3.67 | 16.57 | 20.39 | 27.04 | 23.89 | 14.44 |
| **Median** | 6.55 | 6.31 | 1.79 | 9.10 | 4.38 | 6.41 | 5.71 | 1.43 |
| **St. d.** | 188.42 | 33.66 | 5.06 | 17.56 | 53.69 | 55.54 | 84.77 | 84.57 |
| **Minimum** | 3.48 | 2.77 | 0.68 | 2.32 | 0.58 | 0.42 | 0.69 | 0.36 |
| **Maximum** | 1130.10 | 213.86 | 23.59 | 70.83 | 311.13 | 230.34 | 559.95 | 575.49 |

**Table C.5:** *Samples’ cluster membership, their enrichment factor, and pollution load index (PLI)*

| **Sample No.** | **Cluster membership** | **Enrichment factor** | | | | | | | | **PLI** |
| --- | --- | --- | --- | --- | --- | --- | --- | --- | --- | --- |
|  |  | **Cu** | **Zn** | **Mo** | **Cd** | **Sb** | **Pb** | **Hg** | **As** |  |
| **1** | 1 | 6.69 | 2.77 | 1.56 | 3.82 | 0.86 | 0.42 | 0.69 | 0.36 | 0.39 |
| **3** | 2 | 6.40 | 4.99 | 0.95 | 5.80 | 2.61 | 5.90 | 11.49 | 1.41 | 0.73 |
| **5** | 1 | 20.60 | 35.95 | 23.599 | 70.83 | 15.94 | 98.16 | 10.02 | 8.35 | 5.53 |
| **6** | 2 | 6.01 | 7.08 | 1.63 | 19.90 | 4.48 | 7.38 | 5.37 | 2.43 | 0.59 |
| **7** | 2 | 8.16 | 8.64 | 4.87 | 59.588 | 8.04 | 8.07 | 12.87 | 4.24 | 0.90 |
| **9** | 2 | 5.59 | 55.82 | 1.32 | 24.26 | 7.28 | 11.90 | 8.73 | 1.36 | 1.03 |
| **10** | 2 | 26.96 | 24.97 | 3.90 | 47.66 | 21.45 | 50.97 | 42.89 | 4.47 | 0.47 |
| **11** | 2 | 65.13 | 13.68 | 3.09 | 12.58 | 19.81 | 21.04 | 13.58 | 1.18 | 0.98 |
| **12** | 2 | 14.76 | 9.90 | 3.45 | 21.06 | 9.48 | 13.71 | 11.37 | 2.76 | 0.48 |
| **15** | 2 | 3.48 | 4.45 | 1.81 | 22.09 | 4.97 | 1.75 | 3.98 | 4.56 | 0.21 |
| **16** | 1 | 10.69 | 7.67 | 4.06 | 12.40 | 23.72 | 31.59 | 10.05 | 2.09 | 1.70 |
| **17** | 2 | 279.09 | 7.95 | 2.85 | 23.22 | 26.12 | 25.08 | 14.63 | 1.96 | 1.45 |
| **18** | 2 | 55.03 | 70.44 | 8.62 | 63.18 | 52.12 | 195.56 | 174.37 | 3.95 | 2.34 |
| **19** | 2 | 41.12 | 14.12 | 7.11 | 7.24 | 54.40 | 13.30 | 4.73 | 1.32 | 12.75 |
| **19-1** | 2 | 605.02 | 67.98 | 18.30 | 31.96 | 158.21 | 230.34 | 559.95 | 575.49 | 15.84 |
| **20** | 2 | 1130.10 | 213.86 | 22.53 | 61.19 | 311.13 | 216.87 | 22.03 | 3.44 | 14.56 |
| **21** | 2 | 13.33 | 8.71 | 1.56 | 19.06 | 4.29 | 10.45 | 18.87 | 1.79 | 0.81 |
| **22** | 2 | 6.12 | 4.71 | 4.19 | 8.54 | 5.77 | 6.92 | 13.84 | 2.08 | 0.75 |
| **23** | 2 | 7.73 | 5.92 | 2.62 | 8.01 | 3.61 | 4.54 | 14.42 | 1.50 | 0.68 |
| **24** | 1 | 5.67 | 3.89 | 1.78 | 3.62 | 0.82 | 1.86 | 3.91 | 1.36 | 0.74 |
| **26** | 2 | 12.03 | 12.85 | 2.80 | 17.09 | 7.69 | 11.94 | 21.53 | 2.24 | 0.57 |
| **28** | 2 | 5.29 | 4.02 | 1.36 | 8.31 | 1.87 | 3.47 | 5.98 | 1.25 | 0.43 |
| **29** | 1 | 13.22 | 7.52 | 3.39 | 13.83 | 135.311 | 129.222 | 13.69 | 1.81 | 2.34 |
| **30** | 1 | 3.80 | 5.83 | 3.67 | 22.45 | 1.68 | 3.17 | 5.39 | 1.82 | 0.62 |
| **33** | 2 | 8.56 | 12.41 | 2.99 | 9.15 | 2.06 | 4.79 | 14.82 | 2.40 | 0.68 |
| **36** | 1 | 5.42 | 3.48 | 1.22 | 2.98 | 0.67 | 1.56 | 3.22 | 0.89 | 0.73 |
| **38** | 1 | 5.41 | 4.03 | 1.32 | 5.36 | 1.21 | 2.44 | 4.82 | 1.41 | 0.57 |
| **42** | 1 | 6.00 | 4.41 | 1.31 | 8.01 | 1.80 | 4.14 | 8.65 | 1.35 | 0.98 |
| **44** | 1 | 5.47 | 4.05 | 1.18 | 3.59 | 1.62 | 3.33 | 11.00 | 1.21 | 0.93 |
| **46** | 1 | 5.43 | 3.85 | 1.52 | 3.73 | 0.84 | 2.23 | 4.02 | 0.91 | 0.69 |
| **48** | 1 | 11.82 | 6.69 | 3.70 | 9.06 | 2.04 | 1.58 | 1.63 | 1.02 | 0.41 |
| **49** | 1 | 6.40 | 3.94 | 1.05 | 3.22 | 1.45 | 2.32 | 2.32 | 1.45 | 0.81 |
| **50** | 1 | 4.90 | 2.96 | 0.95 | 2.32 | 1.04 | 1.89 | 0.84 | 1.00 | 0.79 |
| **51** | 1 | 5.34 | 3.85 | 1.26 | 2.56 | 0.58 | 1.88 | 1.84 | 0.96 | 0.80 |
| **53** | 1 | 5.42 | 4.48 | 1.17 | 4.77 | 1.07 | 2.30 | 1.72 | 0.98 | 0.51 |
| **54** | 1 | 5.95 | 4.59 | 0.91 | 3.71 | 0.84 | 3.52 | 2.67 | 1.04 | 0.69 |
| **55** | 1 | 5.24 | 3.30 | 0.84 | 3.42 | 0.77 | 1.68 | 1.85 | 1.28 | 0.61 |
| **56** | 2 | 8.17 | 6.99 | 3.32 | 20.27 | 3.04 | 5.47 | 4.87 | 1.90 | 0.87 |
| **57** | 2 | 9.04 | 7.11 | 1.73 | 10.59 | 10.72 | 33.87 | 4.77 | 1.09 | 1.30 |
| **58** | 2 | 9.68 | 9.46 | 2.74 | 26.83 | 4.53 | 10.58 | 6.04 | 2.26 | 1.12 |

| **Field No.** | **Cluster membership** | **Enrichment factor** | | | | | | | | **PLI** |
| --- | --- | --- | --- | --- | --- | --- | --- | --- | --- | --- |
|  |  | **Cu** | **Zn** | **Mo** | **Cd** | **Sb** | **Pb** | **Hg** | **As** |  |
| **58Ph** | 2 | 11.40 | 9.02 | 4.32 | 22.64 | 5.09 | 10.83 | 5.43 | 2.69 | 1.08 |
| **59** | 2 | 7.32 | 6.72 | 2.12 | 17.25 | 5.82 | 10.41 | 7.76 | 2.10 | 0.78 |
| **60** | 1 | 5.25 | 3.88 | 1.43 | 2.92 | 0.66 | 3.54 | 1.58 | 1.26 | 0.80 |
| **61** | 1 | 5.21 | 3.63 | 0.68 | 5.52 | 0.62 | 3.08 | 1.99 | 1.24 | 0.83 |
| **62** | 1 | 5.73 | 4.69 | 1.13 | 3.46 | 4.67 | 9.41 | 1.24 | 1.23 | 0.97 |
| **66** | 1 | 1.04 | 3.17 | 4.99 | 19.44 | 1.71 | 1.31 | 1.71 | 0.00 | 1.23 |


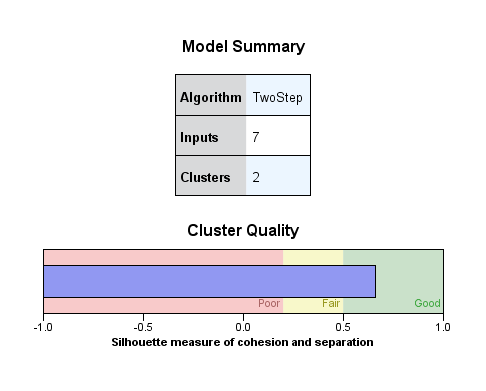

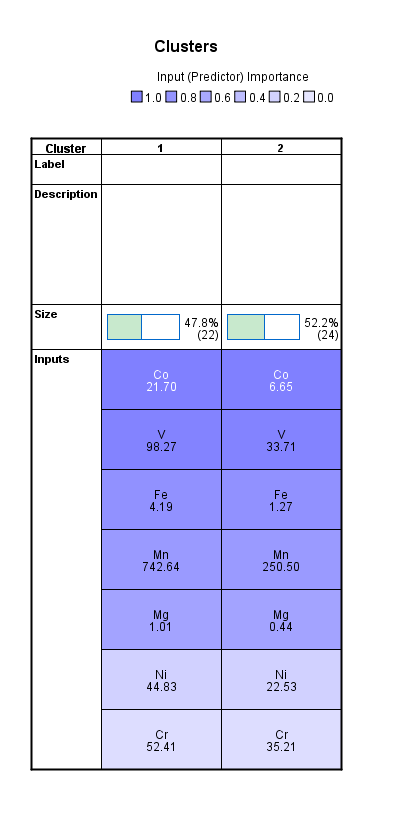


**Figure C.1:** Model summary of two-step cluster analysis (left), and the obtained clusters with the average concentration of the distinguishing elements with their importance in the two-step clustering model (Right).


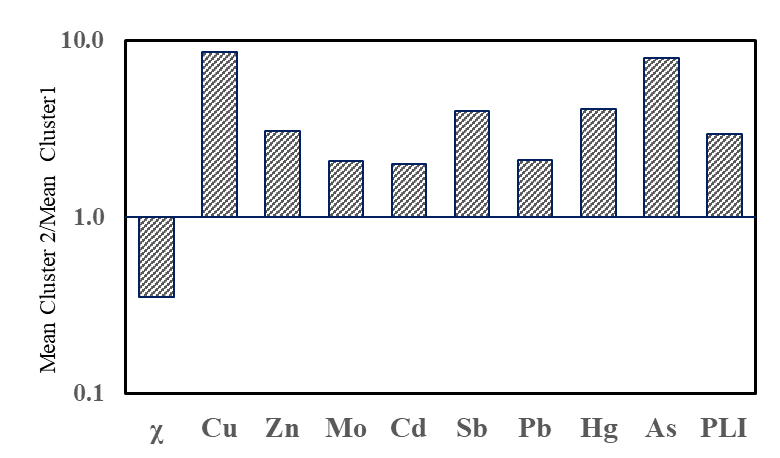


**Figure C.2:** HMs mean concentrations in cluster 2 compared to cluster 1.
